# Natural language processing and machine learning algorithm to identify brain MRI reports with acute ischemic stroke

**DOI:** 10.1371/journal.pone.0212778

**Published:** 2019-02-28

**Authors:** Chulho Kim, Vivienne Zhu, Jihad Obeid, Leslie Lenert

**Affiliations:** 1 Department of Neurology, Hallym University College of Medicine, Chuncheon, Korea; 2 Medical University of South Carolina, Charleston, South Carolina, United States of America; 3 Biomedical Informatics Center, Medical University of South Carolina, Charleston, South Carolina, United States of America; 4 Department of Internal Medicine, Medical University of South Carolina, Charleston, South Carolina, United States of America; University College London, UNITED STATES

## Abstract

**Background and purpose:**

This project assessed performance of natural language processing (NLP) and machine learning (ML) algorithms for classification of brain MRI radiology reports into acute ischemic stroke (AIS) and non-AIS phenotypes.

**Materials and methods:**

All brain MRI reports from a single academic institution over a two year period were randomly divided into 2 groups for ML: training (70%) and testing (30%). Using “quanteda” NLP package, all text data were parsed into tokens to create the data frequency matrix. Ten-fold cross-validation was applied for bias correction of the training set. Labeling for AIS was performed manually, identifying clinical notes. We applied binary logistic regression, naïve Bayesian classification, single decision tree, and support vector machine for the binary classifiers, and we assessed performance of the algorithms by F1-measure. We also assessed how n-grams or term frequency-inverse document frequency weighting affected the performance of the algorithms.

**Results:**

Of all 3,204 brain MRI documents, 432 (14.3%) were labeled as AIS. AIS documents were longer in character length than those of non-AIS (median [interquartile range]; 551 [377–681] vs. 309 [164–396]). Of all ML algorithms, single decision tree had the highest F1-measure (93.2) and accuracy (98.0%). Adding bigrams to the ML model improved F1-mesaure of naïve Bayesian classification, but not in others, and term frequency-inverse document frequency weighting to data frequency matrix did not show any additional performance improvements.

**Conclusions:**

Supervised ML based NLP algorithms are useful for automatic classification of brain MRI reports for identification of AIS patients. Single decision tree was the best classifier to identify brain MRI reports with AIS.

## Introduction

Stroke is one of the leading causes of death and morbidity worldwide, and a major health problem according to the Global Burden of Disease study [1, 2]. When estimating the burden of a stroke, the incidence, prevalence, and disability-adjusted life-years (DALYs) of the stroke are combined [1, 3]. However, in most studies, the incidence of stroke is not a true national-level figure, but estimated figures that were taken into account in large-scale population-based cohort study results [4, 5]. Alternately, electronic health records can be used to estimate acute stroke incidence [6, 7]. The medical record contains laboratory data, clinical information, and the International Classification of Diseases (ICD) diagnosis codes. Those codes can simply indicate whether a patient has been admitted for a stroke, but often they cannot accurately distinguish whether the patient was hospitalized for acute symptoms of stroke or other problems arising from stroke [8]. However, through various MRI imaging techniques, we can confirm whether the stroke is ischemic or hemorrhagic, and whether it is acute or chronic [9] In addition, MRI reports are rarely coded at a report reading level, and unstructured data such as text reports and imaging data often contains useful information.

One approach for unlocking the information in text descriptions of MRI readings is natural language processing (NLP). NLP has been actively studied in analyses of unstructured text data, which accounts for a large portion of the medical records such as admission notes, nursing records and discharge summaries [10, 11]. NLP tools can be applied in a rule-based fashion to parse out the meaning of texts, although they are employing both supervised and unsupervised machine learning (ML) algorithms [12] Prior stroke research includes feasibility studies of NLP for predicting a future stroke [13], extracting risk factor information [14], and timely screening for urgent thrombolysis [15]. In addition, several reports have used NLP to predict the progression of cancer or to classify breast pathology by analyzing free text radiology reports [16, 17]. However, no NLP study has occurred to identify patients with acute ischemic stroke (AIS) from radiologic reports of brain MRIs. Our aims were to implement ML algorithms that can automatically identify AIS patients based on the free-text in the patients’ brain MRI reports. In addition, we compared the performances of different supervised ML algorithms with a harmonized mean of precision and recall in this classification task.

## Materials and methods

### Participants and MRI sampling

This is a single center retrospective case control study. The study protocol was approved by the Institutional Review Boards and Ethics Committee at Chuncheon Sacred Heart Hospital (IRB No. 2017–114), with a waiver of informed consent. Our hospital stores entire medical records in a clinical data warehouse, which allowed us to screen all brain MRI reports performed between January 1, 2015 and December 31, 2016. We identified MRI reports that included the conventional stroke MRI sequence. Conventional stroke MRI sequences were T2-weighted image, fluid-attenuated inversion recovery, gradient echo image, diffusion weighted image, apparent diffusion coefficient map and non-contrast time-of-flight magnetic resonance (MR) angiography. MRI reports, which also included perfusion or contrast-enhanced MR sequence, were not excluded from the sample. If a patient had a sequence of multiple MRI examinations of the brain, only the first brain MRIs in for each patient was included. During the study period, one neuroradiologist read all brain MRI images. At the time of MRI reading, the neuroradiologist could access information about the chief complaint or reason for referral of the patients to propose an impression of the reading. Additionally, outside imaging or a past imaging were available for the patient, those images were used as a reference for reading the current brain MRI image.

### Annotation of MRI reports

The format of the brain MRI reading is depicted in S1 Fig. All the reports were in English. Of these reports, we collected only text data on the radiologists’ descriptions and findings of brain MRI reports, and we specifically excluded the texts on the report’s conclusions. We consecutively enrolled patients who were admitted to the hospital within 7 days of neurological symptom onset, had consented to participate in a research registry, and were diagnosed with AIS both clinically and radiologically. The registry contains demographic variables, laboratory data, radiologic lesion information, and all the information related to stroke from symptom to post-discharge, such as onset time, emergency department visit time, stroke subtype, type of acute treatment, early neurologic deterioration, and 3-month functional outcome [18]. However, the neuroradiologist could not access the registry which included consensus information about whether the patient had AIS. The gold standard labeling of AIS relied on previous diagnosis of AIS in a prospective AIS registry. In the registry, ischemic stroke was defined as having the relevant lesion on MRI and acute neurological symptoms lasting more than 24 hours [19]. All brain MR images, which were performed in non-AIS subjects and included more than stroke MR sequences, were used as control groups when comparing the text in the findings section of the reading. The control group included patients who underwent brain MRI for a specific disease, such as brain tumor or intracranial hemorrhage, as well as those who underwent MRI as a health check-up or outpatient evaluation for specific symptoms such as headache or dizziness.

### NLP algorithm

**Fig 1:**
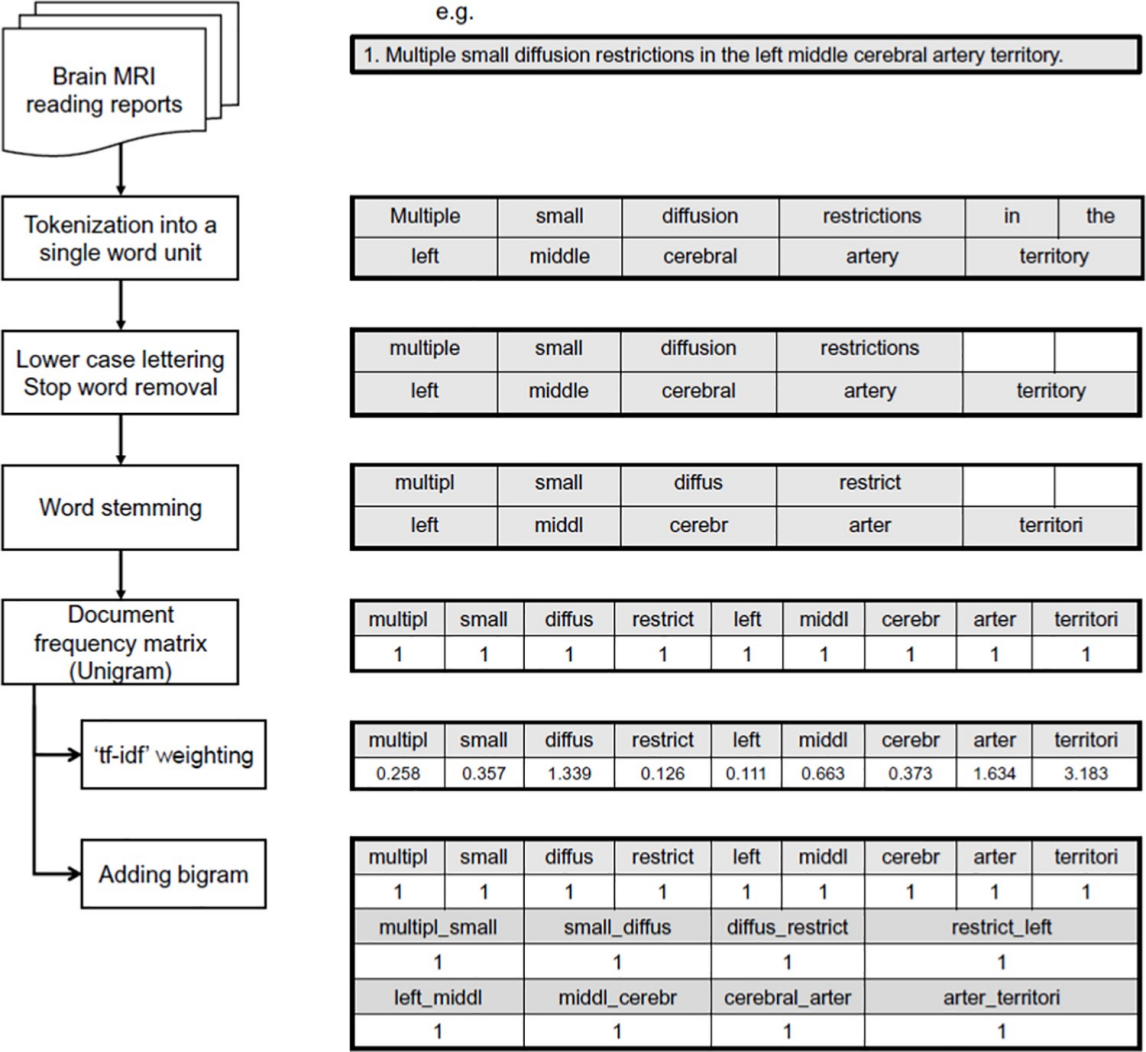
Preprocessing flow chart of “quanteda” natural language processing package.

We used the open source “quanteda” R package, which classifies texts into 2 groups using NLP algorithms (Fig 1) [20]. In brief, full text brain MRI reading sentences were initially parsed into “tokens,” with numbers, punctuations, symbols and hyphens in the original text data removed. Then, we used lowercase lettering, stop word removal, and word stemming to normalize those data [21]. Finally, we obtained the document-feature matrix (dfm), which is a vector representation of tokens that are truncated from the whole text. We used 4 types of dfm vectorization: unigram, unigram + term frequency-inverse document frequency (tf-idf), adding bigram, and adding bigram + tf-idf. Term frequency (tf) is the number of times that a particular word occurs in a document, and document frequency is the count of documents containing a particular word [22]. Inverse document frequency (idf) is the reciprocal of document frequency. For example, idf value is small for common words such as “the”, and large for those that are not common. Tf-idf is a way of giving weight to a word vector by multiplying tf by idf. Bigram is a two-word vector that is arranged in a sequential manner, which helps to differentiate a document by the word quantity as well as the word order [23].

### Statistical analysis

We performed descriptive analyses of differences between AIS and non-AIS reports. Character lengths of the reports were compared using a Mann-Whitney U test. We used the “keyness plot” to determine which words were frequently used in AIS readings and which words were frequently used in non-AIS. The chi-square value of the plot indicates the frequency of the words appearing in the document, and that value becomes smaller and approaches zero when the words appearing simultaneously in two documents of AIS and non-AIS patients [24].

To classify the two reference standards of AIS and non-AIS, four types of dfm matrix were applied to 4 ML algorithms—binary logistic regression (BLR), naïve Bayesian classification (NBC), single decision tree (SDT) and support vector machine (SVM). We split the text data into training and testing datasets with a ratio of 7:3 and used 10-fold cross-validation to train the models on the training set. We compared the performance of the four algorithms with F1-measure (harmonized mean and precision and recall) and receiver operating characteristic (ROC) curve analysis in classifying AIS and non-AIS reports. The e1071, rpart, and quanteda packages were used to perform all our statistical analyses and ML algorithms; all statistical computing was performed with R (version 3.4.3) [25, 26].

In addition, we performed a qualitative analysis of MRI readings that were misclassified by the best performing ML model. In the case of supervised ML classifiers, it may be important to correct the class imbalance during the training process to reduce the bias and to obtain better performance [27]. Therefore, ML training was performed by random sampling of training data corresponding to each class balanced to 50:50 by setting with a case number (303 vs 303), a control number (1815 vs 1815), or a desired number (5000 in total) [28].

## Results

Of all 8,793 brain MRI readings, 4,238 MRIs included more than conventional stroke MRI sequences. A total of 3,024 MRIs was included in the final analysis, excluding those taken more than twice during the study period. Raw data can be downloaded in the Supporting Information File (S1 File). The mean age of the participants and proportion of female were 60.0 ± 17.6 years and 51.7% (1,563 out of 3,024), respectively. During the study period, there were 469 AIS patients were enrolled in the registry; we excluded 37 subjects with an AIS because they did not have enough stroke MRI sequence images, or they only had MRI images from outside the hospital. The test and training data sets included 432 (14.3%) patients with MRI readings that confirmed AIS. The resulting training dataset had 303 AIS and 1,815 non-AIS reports, and the test dataset had 129 AIS and 777 non-AIS reports.

**Fig 2:**
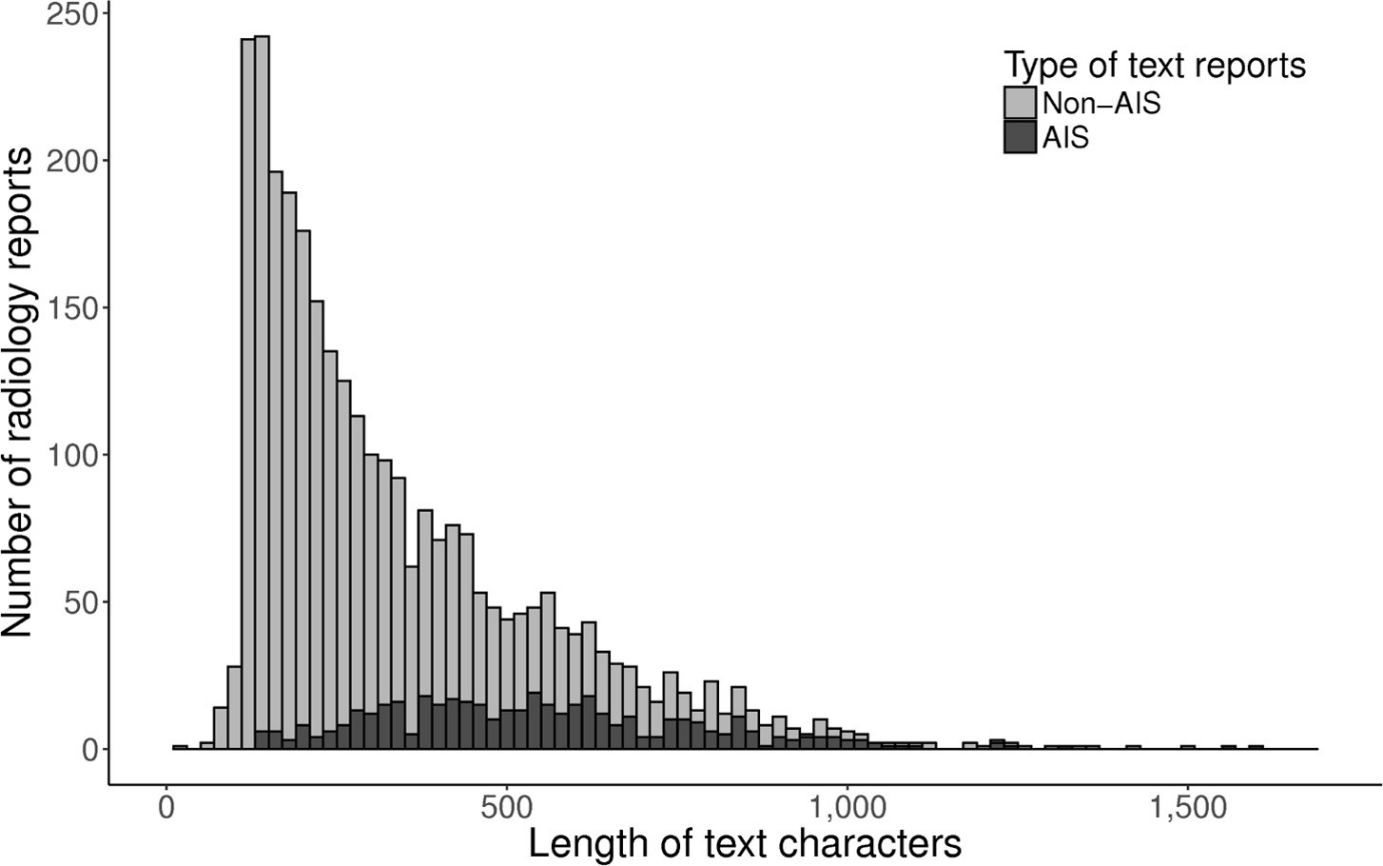
Difference of the text character lengths between AIS and non-AIS reports. AIS, acute ischemic stroke.

**Fig 3:**
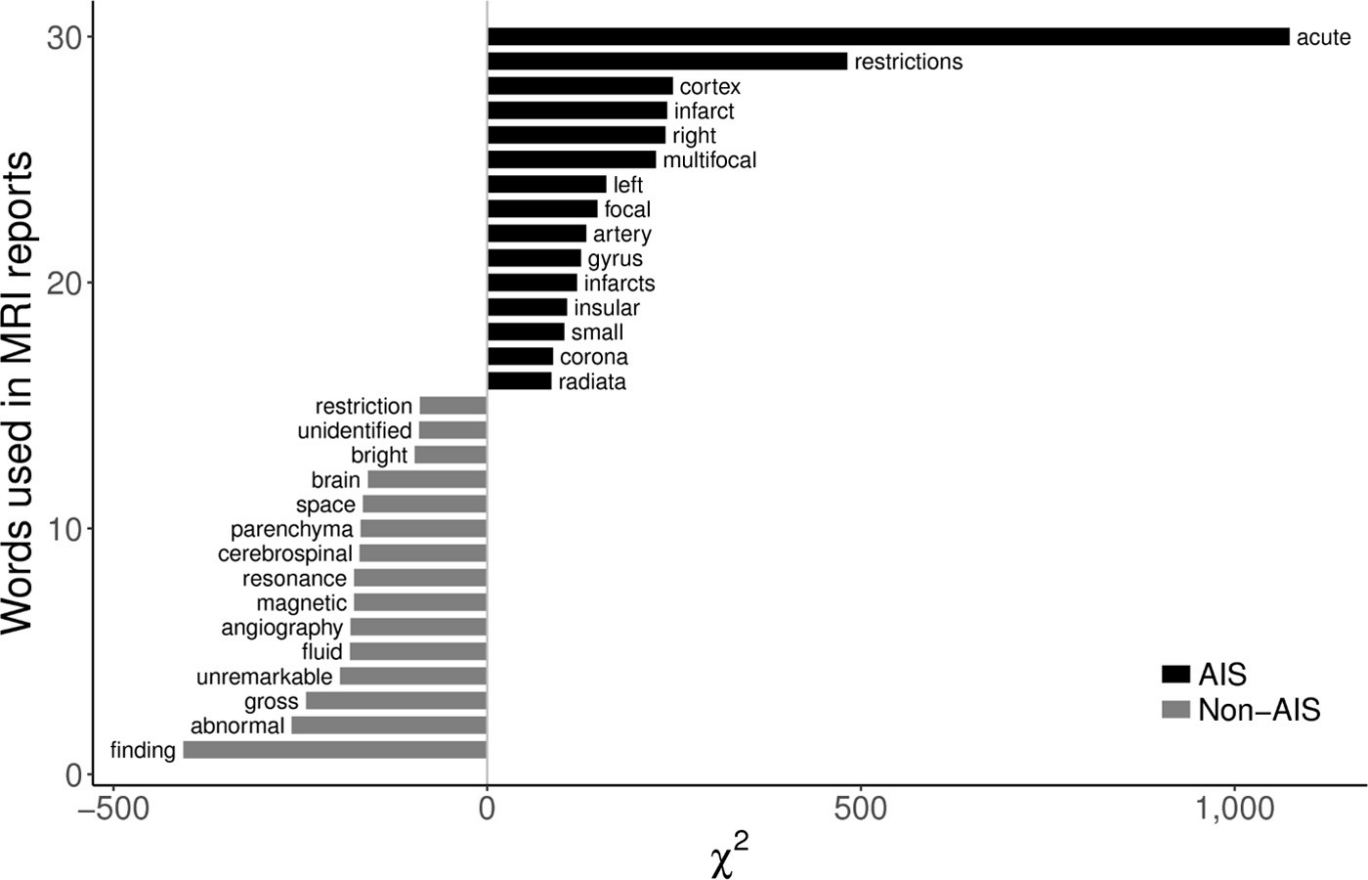
Result of keyness plot analysis of AIS and non-AIS reports. AIS, acute ischemic stroke.

Fig 2 depicts the difference of the text character lengths between AIS and non-AIS reports. MRI reports of AIS patients had a larger amount of text characters versus reports of non-AIS patients (median [interquartile range]; 551 [377–681] vs. 309 [164–396]). We show the 15 most frequently occurring words in the AIS reading and those words in the non-AIS readings, and we summarize them in Fig 3. For example, the word “acute” was used most frequently in AIS reports, followed by “restrictions” and “cortex”. On the other hand, the words “gross”, “abnormal”, and “finding”, which usually represent normal conditions (e.g., “No gross abnormal findings was observed.”), appeared frequently in non-AIS reports.

### MRI reading classification by NLP

Of 2,118 randomly selected reports in the training dataset, text preprocessing of MRI reports yielded 1,146 keyword features after removing numbers, punctuations, symbols, hyphens and stop words. When we extracted the keywords using bigram as well as unigram in text classification, 9,402 features were obtained and entered into the training dataset and used to predict each ML algorithm. Precision, also known as positive predictive value, was defined as the ratio of true positive over true positive plus false positive, while recall, also known as sensitivity, was defined as the ratio of true positive results in the test over the true positive plus false negative. We presented the performance of each algorithms as the F1-measure (harmonized mean of precision and recall):

$$F1\;measure=2\times \frac{precision\times recall}{precision+recall}$$

**Fig 4:**
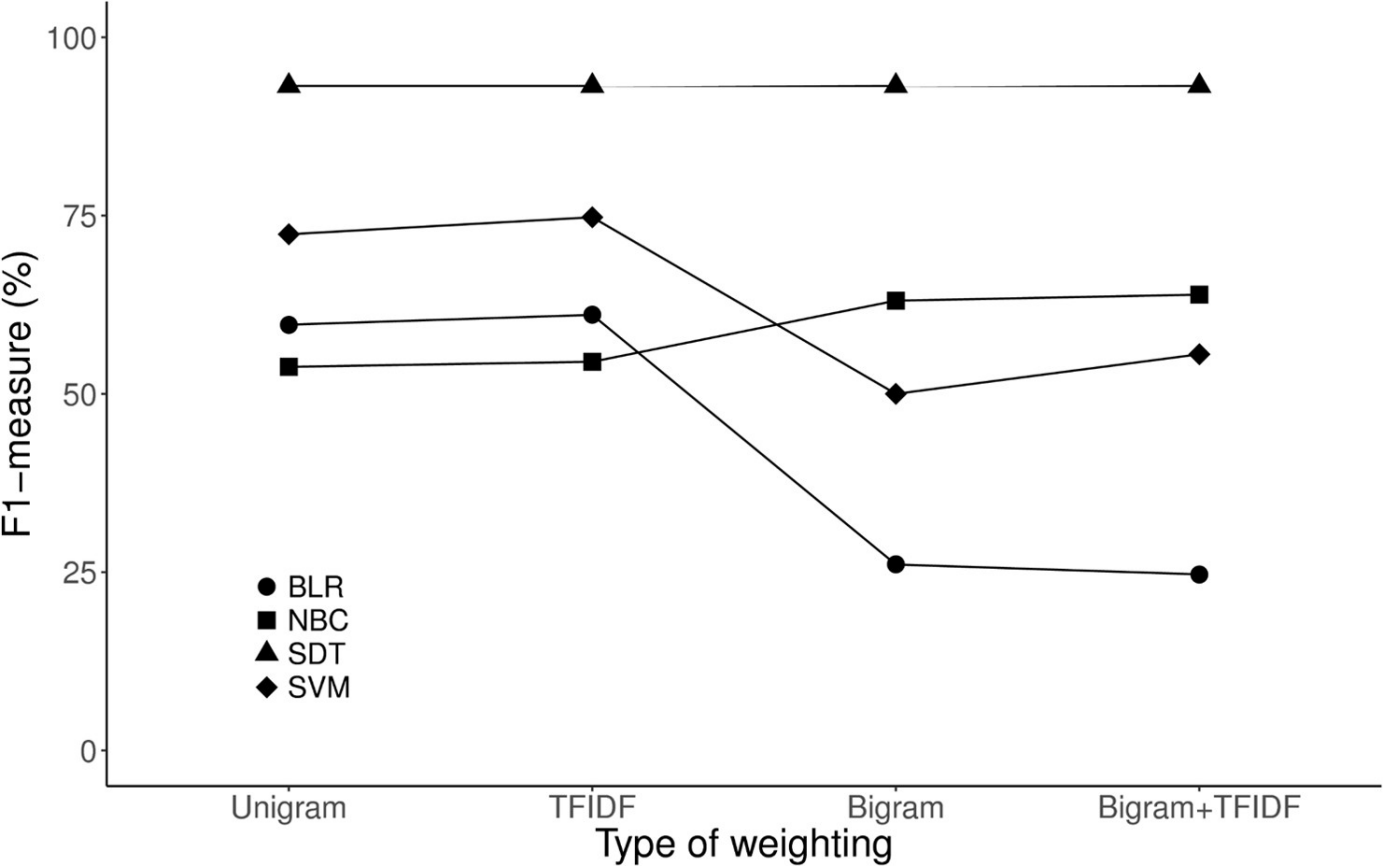
Comparison of ML and NLP algorithms for classifying the brain MRI reports. ML, machine learning; NLP, natural language processing, BLR, binary logistic regression; NBC, naïve Bayesian classification; SDT, single decision tree; SVM, support vector machine; TFIDF, term frequency-inverse document frequency.

Fig 4 shows a comparison of these performance of each algorithm and detailed results are presented in Table 1. Of all the ML algorithms, the F1-measure of SDT was the highest in unigram classification even if we added bigram or tf-idf weights in the ML model. Adding the bigram to the ML model improved performance in NBC, but not in other models. S2 Fig also shows the area under the ROC of each ML algorithm. Adding the bigram to the ML model, which requires more computational efforts in performing the ML task, could improve the recall slightly, but overall performance of the BLR or SVM was not improved.

**Table 1:** Results of performance of each machine learning algorithms.

	TP	FP	FN	TN	Total	Sensitivity (Recall)	Specificity	PPV (Precision)	NPV	Accuracy	F1-measure	P for χ^2^
BLR unigram	100	106	29	671	906	77.5	86.4	48.5	95.9	85.1	59.7	<0.001
BLR tf-idf	102	103	27	674	906	79.1	86.7	49.8	96.1	85.7	61.1	<0.001
BLR adding bigram	64	298	65	479	906	49.6	61.6	17.7	88.1	59.9	26.1	0.020
BLR adding bigram+tf-idf**	60	297	69	480	906	46.5	61.8	16.8	87.4	59.6	24.7	0.082
NBC unigram	110	170	19	607	906	85.3	78.1	39.3	97.0	79.1	53.8	<0.001
NBC tf-idf	112	170	17	607	906	86.8	78.1	39.7	97.3	79.4	54.5	<0.001
NBC adding bigram	111	112	18	665	906	86.0	85.6	49.8	97.4	85.7	63.1	<0.001
NBC adding bigram+tf-idf	116	118	13	659	906	89.9	84.8	49.6	98.1	85.5	63.9	<0.001
SDT unigram*	123	12	6	765	906	95.3	98.5	91.1	99.2	98.0	93.2	<0.001
SDT tf-idf*	123	12	6	765	906	95.3	98.5	91.1	99.2	98.0	93.2	<0.001
SDT adding bigram*	123	12	6	765	906	95.3	98.5	91.1	99.2	98.0	93.2	<0.001
SDT adding bigram+tf-idf*	123	12	6	765	906	95.3	98.5	91.1	99.2	98.0	93.2	<0.001
SVM unigram	76	5	53	772	906	58.9	99.4	93.8	93.6	93.6	72.4	<0.001
SVM tf-idf	80	5	49	772	906	62.0	99.4	94.1	94.0	94.0	74.8	<0.001
SVM adding bigram	43	0	86	777	906	33.3	100.0	100.0	90.0	90.5	50.0	<0.001
SVM adding bigram+tf-idf	50	1	79	776	906	38.8	99.9	98.0	90.8	91.2	55.6	<0.001

TP, true positive; FP, false positive; FN, false negative; TN, true negative; PPV, positive predictive value, NPV, negative predictive value; BLR, binary logistic regression; tf-idf, term frequency-inverse document frequency; NBC, Naïve Bayesian classification; SDT, single decision tree; SVM, support vector machine.

* the best classifiers.

** the worst classifier.

### Decision tree and error analysis

Performance of SDT produced 93.2 of F1-measure as well as a good accuracy (98.0% in Table 1). The”acut” feature was located in the root node, while the “intracerebr” and “intraventricular” features, which usually imply an intracranial hemorrhage, were located in the internal nodes to distinguish AIS from non-AIS. There were 12 false positive and 6 false negative results for this algorithm, and the relevant explanations for the misclassification are summarized in Table 2.

**Table 2:** Error analysis of result of single decision tree in classifying AIS and non-AIS.

Reason for misclassification	FN	FP
Various disease condition could be accompanied with MR diffusion restrictions	3	0
Reading including the recent or old cerebral hemorrhages	3	4
Lesions with diffusion restrictions in MRI but no relevant clinical symptoms	5	0
Miscellaneous	1	2
Total	12	6

AIS, acute ischemic stroke; FN, false negative; FP, false positive.

### Model considering class imbalance of training

The training dataset of the single decision tree was composed of 303 AIS cases and 1,815 controls. We used three methods to resolve the class imbalance in decision tree training: over sampling (1,815 vs 1,815), under sampling (303 vs. 303), and fixed number (n = 5,000) sampling (S1 Table). There was no significant change of performance in precision, recall, accuracy, and the F1-measure when we obtained test results after training with those balanced data sets.

## Discussion

In our study, NLP algorithms were a useful tool to identify patients with the phenotype of AIS, using unstructured radiologic reports of brain MRIs. Interestingly, SDT-based binary classification showed high precision (91.1%) and recall (95.3%), and additional weighting method for dfm did not show further improvement of several ML algorithms. Error analysis of SDT showed that most of the errors were not caused by NLP or ML algorithms but by the MRI imaging characteristics of the AIS itself. In terms of classification imbalance during the SDT training process, there were no significant differences of the F1-measures of ML predictions when we performed training processes using several class-balanced data.

Since the 1980s when CT equipment in conjunction with X-rays began to be used for the diagnosis of human illness, the development of diagnostic equipment has evolved rapidly. Various imaging techniques have been used to diagnose specific brain diseases, and brain MRI has become an essential tool for the diagnosis of various diseases including AIS [29]. Because MRI images are proliferating at a rapid rate and the MRI reading is an unstructured text data, it is becoming increasingly difficult to classify those diagnostic images manually within a fixed time period. Moreover, it may be inaccurate to classify CNS diseases using diagnostic codes such as the ICD [30, 31], which are usually coded manually. In the case of AIS caused by other main diseases, such as cardiogenic AIS caused by acute myocardial infarction, the stroke diagnosis code may be secondary to the ICD codes. In addition, two studies that analyzed a trend of intravenous thrombolysis after acute ischemic stroke with the ICD-9 codes reported that the ICD-9 codes tended to underestimate intravenous thrombolysis [32, 33]. Therefore, diagnostic codes such as the ICD-9 may return inaccurate search results for certain diseases such as AIS. However, our study demonstrated that information related to an AIS diagnosis could be successfully extracted in large numbers of brain MRI radiology reports using open source NLP and ML algorithms. We suggest that these automated supervised ML and NLP algorithms could be beneficial in classifying a vast amount of brain MRI reports automatically and accurately.

Our NLP-based ML technique makes it possible to classify and extract useful information efficiently in a short period of time from a large amount of text reports. Wright et al. used lexicon-based ML classification for extracting diabetes-related information from 2000 clinical progress notes and reported that SVM using a bag-of-words approach was effective in classifying them as 0.96 of AUROC and 0.93 of the F1-score [34]. Hassanpour et al. suggested that simple structured texts could be sufficiently classified with a bag-of-words model and complex structured texts with lexicon-based information retrieval methods [35]. In our analysis, we applied bag-of-words NLP algorithms to identify AIS reports from a large amount of brain MRI radiology reports, and their algorithmic performances were comparable to other study results [34,36,37]. Our result suggest that the brain MRI radiology report is not a complex structured text. However, further study is needed to determine whether the bag-of-words model is more important than the higher order classification system for multi-class classification.

Usually adding bigram features on a bag-of-words unigram model improves the classification performance because the text itself is the sum of the sequential vectors [38]. However, combined unigram-bigram features did not improve classification performance in our analysis. The reasons why this phenomenon occurred are as follows. First, applying bigram to input vectors produces a large amount of input data. In our model, input vector size increased from 1,146 to 9,402 features. Moreover, performance of the ML classifier depends on the trade-off between false positives and false negatives. Therefore, the large number of word vectors created by adding bigram features to NLP may have contributed to a further reduction in performance in binary classification. Grundmeier et al. suggested that removal words with low frequency in each text from a large number of input features could successfully identify long bone fractures in radiology reports [39]. Second, in the SDT structure, the more important predictors are located near the root node [40]. Grundmeier et al. studied the NLP classification adding bigram features to the random forest classifier, which is an ensemble of decision trees. And they showed that unigram features had higher Gini importance values when compared to bigram features [6]. Therefore, we speculate that the performance of SDT did not improve by adding bigram because unigram features were located in the uppermost node in the decision tree.

Fig 3 shows the results of a keyness plot indicating "keyword" features and comparing their differential associations with an AIS versus a non-AIS group. That representative example illustrates that “keyword searching” can extract information but in an inefficient way when compared to the NLP method. A large number of words expressing stroke lesion were identified in the AIS reports, while those that described normal reading, such as “unidentified bright object”, “unremarkable” or “no gross abnormal finding” were located in those of non-AIS. However, the words “restriction” or “restrictions” appeared in both AIS and non-AIS reports. Because word stemming as well as lowercase lettering used in NLP can condense various types of words into a single etymology, it is possible to process text features more efficiently with NLP versus keyword searching in text classification. Doan et al. reported that an NLP tool had a higher sensitivity (93.6% vs. 41.0%) in identifying Kawasaki disease in emergency department notes when compared to a simple keyword research, which suggested that the NLP tool could be a good decision support system for the proper diagnosis in an emergent clinical setting when compared to knowledge-based clinical decision-making alone [41]. Thus, we also showed that text mining using NLP had a high accuracy and efficiency compared to keyword searching.

We found that radiology reports of AIS had a longer length than those of non-AIS. Text length could be an important marker in differentiating ham and spam in supervised text message classification [42]. Several structured data such as age and sex are not included in protected health information identifiers and are readily available from the electronic health record, those structured data contain valuable information related to the risk of developing a particular disease. Therefore, it is expected that additional modeling with unstructured data and selected structured data may have a beneficial effect on the performance of ML algorithms in classifying radiology reports. However, we only used the deidentified unstructured text data for this study; further research is needed to determine the effects of additionally using structured data to assess classification performance.

In our result, we showed that SDT had a higher performance for binary classification than the other ML algorithms. Generally, a decision tree performs well when dealing with discrete or categorical features, while SVM performs well with continuous features [43]. Chen et al. analyzed the performance of an ML algorithm to categorize oncologic response using abdominal CT and MRI reports; those researchers showed that SVM had a higher performance (accuracy = 90.6, F score = 0.81) versus analyses with Bayes point machine, logistic regression, random forest, or neural network [16]. However, the performance of SVM decreased when more than 2,500 features were included in the ML algorithms. We also identified that F1-measure was lower when SVM was performed using an n-gram, which requires more additional features during training, as compared to unigram bag-of-words training.

Also, the performance of SVM is reported to be better than decision tree when classification is performed using imaging data or voice data [44]. Yadav et al. reported that the decision tree showed high performance when binary classification was performed for traumatic brain injury using brain CT readings [45]. Likewise, we found that, to achieve good performance, it may be better to choose decision tree as a classifier if the researchers choose to perform a binary classification using brain or CT or MRI radiology reports. However, the factors affecting the performance of the classifier include the amount of training data, characteristics of those data, and class imbalance, and the type of classifier [43]. Therefore, we should carefully consider characteristics of the data when we select for the ML classifier of NLP algorithms.

The resulting error analysis for SDT was due to the radiological characteristics of disease in the CNS rather than errors in NLP or ML algorithms. Diffusion-restrictive lesion is not only a main MRI characteristic of the AIS lesion, it is also accompanied by hypoxia, excitotoxicity, and perihematomal ischemia of the brain [46]. Other NLP tools such as continuous skip-gram of word2vec [47] and GloVe [48] could take into account order and proximity of the words. It is worth investigating whether these NLP methodologies can reduce the errors seen in our results.

There are several limitations to our study. First, our text corpus was created at a single institution, and therefore, it is not possible to generalize our findings. However, generalizable results could occur if we use those NLP and ML tools for inter-institutional validation in a future study. Second, we only included brain MRI reports with conventional stroke MRI sequence. In clinical practice, full conventional brain MRI sequence could vary depending on the degree of emergency in a given situation, the patient’s condition, and the laboratory results. In other words, diffusion only MRI instead of the full stroke MRI sequences would be performed in cases of emergency or when a patient is unstable. Although a diffusion only MRI report is sometimes used to diagnose AIS, that technique does not have all the text features of AIS because the report only includes the description of the diffusion MRI. Therefore, it is important to investigate the characteristics of each institutional radiology report before application of NLP and ML algorithms. Lastly, the performance of ML classifiers could be affected by the class proportions in the training dataset [49]. The proportion of brain MRI reporting in AIS may vary significantly depending on the characteristics of each hospital. However, we obtained results using a balanced dataset, so we can expect that differences in class proportion in the training dataset will not affect the outcome.

## Conclusions

Supervised ML and NLP algorithms can successfully classify brain MRI reports for identification of AIS patients. Moreover, these techniques are rapidly developing fields that can automatically classify a vast amount of medical images using deep learning algorithms. However, labeling for the image data is also a challenging problem in the field of image classification. Therefore, the NLP algorithms can be used to label image data for deep learning.

## Supporting information

S1 Fig Format of the brain MRI readings. (DOCX)

S1 File Raw data. (CSV)

S2 Fig ROC curve analysis for ML classifier according to NLP weighting methods. (DOCX)

S1 Table Results of single decision tree for binary classification considering random sampling of training dataset for reducing class imbalance. (DOCX)
