# Supplementary figures and images for: Natural language processing and machine learning algorithm to identify brain MRI reports with acute ischemic stroke
Source: PLoS One. 2019 Feb 28;14(2):e0212778. doi: 10.1371/journal.pone.0212778 (PMC6394972; doi:10.1371/journal.pone.0212778)

**S2 Fig. ROC curve analysis for ML classifier according to NLP weighting methods.**

**
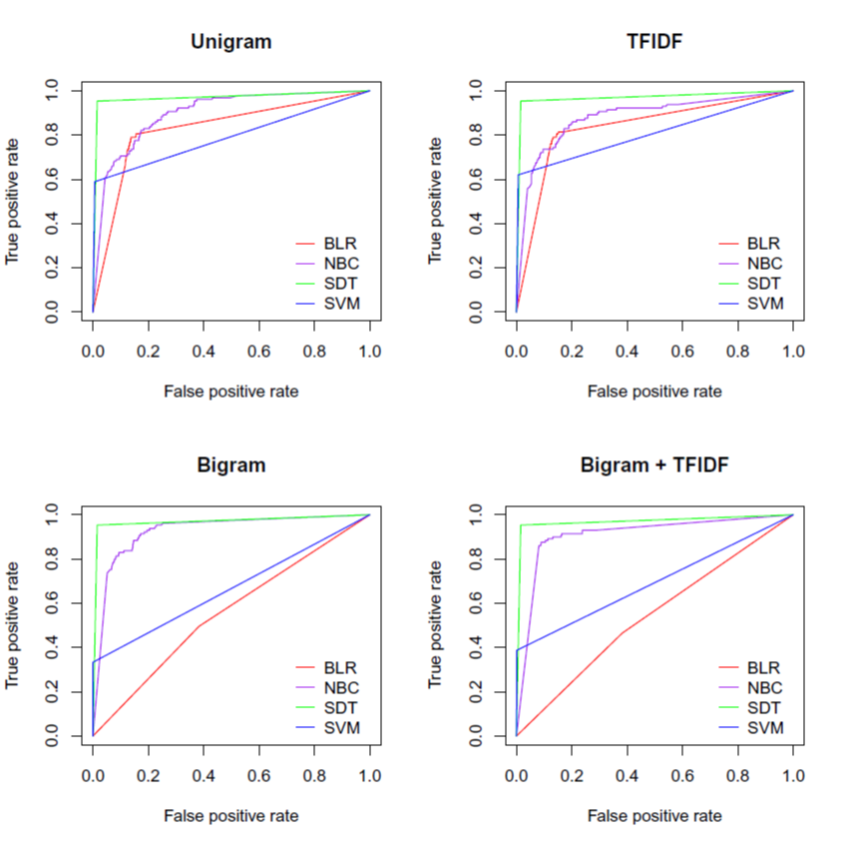
**

Supplement: S2 Fig — (DOCX) [file pone.0212778.s003.docx]
