# Supplementary material for: Natural language processing and machine learning algorithm to identify brain MRI reports with acute ischemic stroke
Source: PLoS One. 2019 Feb 28;14(2):e0212778. doi: 10.1371/journal.pone.0212778 (PMC6394972; doi:10.1371/journal.pone.0212778)
Supplement: S1 Table — (DOCX) [file pone.0212778.s004.docx]

**S1 Table. Results of single decision tree for binary classification considering random sampling of training dataset for reducing class imbalance.**

|  |  | Case | Control | TP | FN | FP | TN | Total | Sensitivity (Recall) | Specificity | PPV (Precision) | NPV | Accuracy | F1-measure |
| --- | --- | --- | --- | --- | --- | --- | --- | --- | --- | --- | --- | --- | --- | --- |
| Original | training | 303 | 1,815 | 295 | 8 | 29 | 1,786 | 2,118 | 97.4 | 98.4 | 91.0 | 99.6 | 98.3 | 94.1 |
|  | testing | 129 | 777 | 123 | 6 | 12 | 765 | 906 | 95.3 | 98.5 | 91.1 | 99.2 | 98.0 | 93.2 |
| Over-sampling | training | 1,815 | 1,815 | 1,776 | 39 | 43 | 1,772 | 3,630 | 97.9 | 97.6 | 97.6 | 97.8 | 97.7 | 97.7 |
|  | testing | 129 | 777 | 124 | 5 | 15 | 762 | 906 | 96.1 | 98.1 | 89.2 | 99.3 | 97.8 | 92.5 |
| Under-sampling | training | 303 | 303 | 296 | 7 | 11 | 292 | 606 | 97.7 | 96.4 | 96.4 | 97.7 | 97.0 | 97.0 |
|  | testing | 129 | 777 | 125 | 4 | 24 | 753 | 906 | 96.9 | 96.9 | 83.9 | 99.5 | 96.9 | 89.9 |
| Fixed number | training | 2,492 | 2,508 | 2,431 | 61 | 59 | 2,449 | 5,000 | 97.6 | 97.6 | 97.6 | 97.6 | 97.6 | 97.6 |
|  | testing | 129 | 777 | 124 | 5 | 15 | 762 | 906 | 96.1 | 98.1 | 89.2 | 99.3 | 97.8 | 92.5 |

TP, true positive; FN, false negative; FP, false positive; TN, true negative; PPV, positive predictive value; NPV, negative predictive value.
